# Diaphragmatic Muscle Thickness: A Complementary Tool in Assessing Lung Involvement in Patients with Systemic Sclerosis

**DOI:** 10.3390/medsci14020285

**Published:** 2026-06-01

**Authors:** Claudia Oana Cobilinschi, Florentina Negoi, Elena Icătoiu, Simona Caraiola, Anca Bobircă, Ciprian Jurcuț, Sorin Constatinescu, Emanuel Moisă, Andra Rodica Bălănescu, Radu Dumitru, Daniela Opriș-Belinski, Narcis Copcă

**Affiliations:** 1 Faculty of Medicine, Carol Davila University of Medicine and Pharmacy, 050474 Bucharest, Romania; florentina.negoi@drd.umfcd.ro (F.N.); elena.juganaru@drd.umfcd.ro (E.I.); simona.caraiola@umfcd.ro (S.C.); anca.bobirca@umfcd.ro (A.B.); sorin.constantinescu@umfcd.ro (S.C.); emanuel.moisa@umfcd.ro (E.M.); andra.balanescu@umfcd.ro (A.R.B.); daniela.opris@umfcd.ro (D.O.-B.); narcis.copca@umfcd.ro (N.C.); 2 Department of Rheumatology, Sf Maria Clinical Hospital Bucharest, 011172 Bucharest, Romania; 3 Department of Internal Medicine, Colentina Clinical Hospital, 020125 Bucharest, Romania; 4 Department of Internal Medicine and Rheumatology, Dr. I. Cantacuzino Clinical Hospital, 011437 Bucharest, Romania; 5 Internal Medicine Department, Dr. Carol Davila Central University Emergency Military Hospital, 010825 Bucharest, Romania; cjurcut@gmail.com; 6 Department of Radiology and Medical Imaging, V Babes Institute, Bucharest 050096, Romania; 7 Clinic of Anesthesia and Intensive Care Medicine, Elias Emergency University Hospital, 011461 Bucharest, Romania; 8 Department of Radiology and Medical Imaging, Sf Maria Clinical Hospital Bucharest, 011172 Bucharest, Romania; 9 Department of General Surgery II, Sf Maria Clinical Hospital Bucharest, 011172 Bucharest, Romania

**Keywords:** diaphragm, systemic sclerosis, HRCT

## Abstract

**Background:** Systemic sclerosis (SSc) is an autoimmune disease with interstitial lung disease (ILD) representing the leading cause of mortality. Diaphragmatic muscle impairment, which may contribute to respiratory dysfunction, is underexplored in SSc. **Objective:** This study aimed to assess diaphragmatic thickness using HRCT in patients with SSc-ILD and to investigate whether this parameter correlates with disease severity and clinical outcomes. **Methods:** We conducted a multicentric retrospective observational study that included 161 participants: 69 with SSc-ILD and 92 matched controls without pulmonary disease. Preliminary findings from this cohort were previously communicated as a conference abstract at EULAR 2024. Diaphragmatic thickness was measured on axial and coronal CT images at the celiac axis level by two independent radiologists. Clinical and functional parameters were collected, including forced vital capacity (FVC), diffusing capacity for carbon monoxide (DLCO), echocardiographic findings, and 6 min walking test performance. **Results:** The left hemidiaphragm was significantly thinner in patients with SSc-ILD compared with controls (2.98 ± 1.04 mm vs. 3.44 ± 0.97 mm; *p* = 0.004), while the right hemidiaphragm showed a non-significant trend toward reduction (3.21 ± 1.09 mm vs. 3.52 ± 1.05 mm; *p* = 0.072). Thinning of the right hemidiaphragm correlated significantly with lower FVC (*p* < 0.05). ROC analysis identified an optimal left diaphragm cut-off of ≤3.115 mm (AUC = 0.635, 95% CI: 0.556–0.709, sensitivity = 66.7%, specificity = 60.9%). No associations were found with the autoantibody profile, disease duration, or treatment. Interobserver reliability was excellent (Bland–Altman mean difference −0.002 mm, *p* = 0.95). **Conclusions:** HRCT-measured left hemidiaphragm thickness is significantly reduced in patients with SSc-ILD compared with controls, and right hemidiaphragm thickness correlates with impaired lung function. Although its discriminative performance is modest (AUC 0.635), this parameter may serve as a supplementary zero-burden addition to routine HRCT evaluation alongside pulmonary function tests. The retrospective cross-sectional design precludes conclusions about causality or prognosis; prospective studies incorporating concurrent functional diaphragm assessment are warranted.

## 1. Introduction

Systemic sclerosis (SSc) is a complex autoimmune disease targeting the skin and multiple organs through inflammation, accelerated fibrosis and mixed vasculopathy [1]. According to skin changes, limited or diffuse SSc are disease forms with distinct autoantibody profiles that predict organ involvement, long-term prognosis and the type of damage accrual. When no suggestive skin modification occurs, the subtype is called SSc sine scleroderma [2].

SSc classification criteria from 2013 assign points for key findings such as skin (thickening, scars or digital ulcerations, telangiectasia), vascular (Raynaud’s), lung (interstitial lung disease (ILD), pulmonary arterial hypertension (PAH) and SSc-related autoantibodies [3].

Given the extent of visceral involvement that can dominate during the disease course, research in the last decade has brought focus to tools that can identify early disease and pre-clinical stages to improve patients’ outcome.

Lung involvement in systemic sclerosis: current approach

Among the various organ manifestations of SSc, pulmonary involvement has emerged as a primary focus of clinical research and therapeutic development, given that ILD drives the majority of disease-related deaths despite current treatment advances. Interestingly, while HRCT reveals subclinical interstitial abnormalities in more than 80% of patients with SSc—a finding corroborated by autopsy data—fewer than one third present with clinically evident ILD or SSc-associated ILD (SSc-ILD) [4]. The 10-year mortality rate reaches approximately 40% [5].

Screening for ILD is essential in patients with SSc, starting with the history and physical examination detecting dry cough, dyspnea or Velcro crackles on auscultation, and plain chest X-ray (which can be non-specific in early stages) [6].

Pulmonary function tests (PFTs) can display no abnormalities at disease onset but later reveal a restrictive model with lower forced vital capacity (FVC) and further low diffusing capacity for carbon monoxide (DLCO) when PAH overlaps [7].

HRCT remains the reference imaging modality for SSc-related ILD, characteristically demonstrating a pattern of non-specific interstitial pneumonia (NSIP) with basal-predominant ground-glass opacities, reticulation and traction bronchiectasis [8]. In a lesser proportion of patients, honeycombing is present, indicating a usual interstitial pneumonia (UIP) pattern [9].

Lung ultrasound (LUS) has gained traction as a low-cost radiation-free screening approach for SSc-ILD, with emerging data supporting its utility even in asymptomatic patients [10,11]. Characteristic sonographic findings include B-line artefacts and pleural line irregularities, and their number has been shown to correlate with the extent of fibrotic involvement in HRCT [12].

Muscle involvement in systemic sclerosis: current approach

The incidence of muscle involvement in SSc is between 5 to 96%, 37% in the diffuse form and 23% in the limited form, according to EUSTAR [13]. The antibody profile can help in the diagnosis process as some can correlate with the occurrence of inflammatory myopathies such as topoisomerase I, anti-Ku, anti-U3 RNP, and anti-PM/Scl in overlapping settings. Regarding pulmonary fibrosis, patients with overlap syndrome SSc/polymyositis are more likely to develop it [14]. Although muscle impairment in SSc involves the skeletal muscle, it can also involve respiratory muscles such as the diaphragm [15].

Patients with SSc can frequently present malnutrition due to multiple causes such as gastrointestinal involvement, ongoing inflammation due to disease pathogenesis or immunosuppressive treatment [16].

Sarcopenia is a frequent finding in patients with systemic sclerosis (SSc) and is associated with reduced muscle strength, impaired physical performance, and altered body composition with a low body mass index (BMI). Early identification of sarcopenia may allow the implementation of preventive strategies aimed at reducing disability, preserving functional capacity and better treatment response [17].

Diaphragm evaluation

The diaphragm plays a critical part in the respiratory function, accounting for over 70% of minute ventilation [18]. It consists of two parts—the crural and the costal part—and it separates the chest wall from the abdomen [19].

Diaphragmatic function may be impaired and lead to partial or total loss of muscle force, secondary to multiple pathologies, including autoimmune diseases, such as SSc.

Muscle waste is under investigated in SSc and it can have profound effects on respiratory function. Inflammation, vasculopathy and fibrosis promote muscle degradation including respiratory muscles such as the diaphragm. On the other hand, pulmonary involvement of SSc, namely ILD and PAH, can exacerbate the diaphragmatic weakness, adding to respiratory distress [20]. As muscle wasting progresses in patients with SSc, the combination of fibrotic lung involvement and impaired respiratory muscle function leads to reduced pulmonary compliance and difficulties in maintaining adequate ventilation [21].

Despite its importance, diaphragmatic function is seldom assessed in clinical practice, and the prevalence of diaphragmatic dysfunction, which can contribute to pulmonary symptoms such as dyspnea and cough, is not well-researched [22].

There are various techniques such as fluoroscopy, electromyography, and manometry invasive procedures that are rarely used in clinical practice [23]. Chest X-ray can be used in patients with complete loss of muscle force (diaphragm paralysis) as it may show an elevated hemidiaphragm [24].

In the last years a few studies have shown the role of using ultrasound (US) in identifying diaphragm dysfunction. This is an easy, accessible and non-invasive method, but it has some limitations such as decreased visibility in obese patients and operator dependence [25,26]. In patients with SSc-ILD, we use computed tomography (CT) to help identify and describe the specific pulmonary lesions, but it can also help give a more comprehensive detailing of the diaphragm and give more specific measurements. Previous studies have proven that a HRCT can show a thinning of the diaphragm pillars by measuring at the level of the celiac artery and the first lumbar vertebrae in unilateral diaphragm paralysis [27].

Our primary goal of this study was to evaluate diaphragmatic thickness through HRCT in patients with SSc-ILD and to further determine whether this measurement can become a severity disease predictor. The secondary goal was to investigate correlations between diaphragmatic parameters and other relevant clinical and paraclinical markers in patients with SSc, such as lung function tests, Modified Medical Research Council dyspnea scale (mMRC), modified Rodnan skin score (mRSS), autoantibodies, and other organ involvement.

## 2. Materials and Methods

This retrospective multicentric observational study was designed and reported in accordance with the STROBE statement. Ethical approval was obtained from the Ethics Committee of Sfânta Maria Clinical Hospital, Bucharest, Romania.

Patients were recruited from four major tertiary rheumatology and internal medicine centers in Bucharest: Sf Maria Clinical Hospital, Colentina Clinical Hospital, Dr. Ion Cantacuzino Clinical Hospital, and Dr. Carol Davila Central Military Emergency University Hospital. Eligibility required an established SSc diagnosis meeting ACR/EULAR 2013 classification criteria [3] and a known SSc-ILD pattern through previous CT imaging.

We excluded from our final study group patients diagnosed with SSc but with no pulmonary involvement, patients with overlap syndrome (a condition that satisfies the classification criteria of at least two pathologies such as SSc, inflammatory myopathies, Sjogren’s disease, systemic lupus erythematosus, rheumatoid arthritis), patients with cachexia, neoplastic pathologies and with chronic glucocorticoid treatment dealing with potential secondary myopathy.

ILD confirmation required a comprehensive work-up, including clinical history, physical examination, pulmonary function testing, chest radiography, and HRCT demonstrating patterns consistent with NSIP or UIP. A Warrick score ≥ 7 defined radiologically significant ILD [23].

To conduct our study, we also created a control study group made of adult patients with no previous history of lung impairment or autoimmune disease and who underwent thoracic CT for another medical condition.

To ensure the compatibility of the groups, we efficiently applied the inclusion and exclusion criteria and selected age- and gender-matched patients.

Measurements of diaphragmatic thickness were evaluated by two independent radiologists using only the most recent follow-up CT performed on our included patients. The celiac axis was used as a reference point and, at this level, diaphragm muscle thickness was measured on the axial as well as on the coronal images. Mean obtained values and differences were used for the final analysis.

Demographic data, disease history, clinical, laboratory and imaging parameters were collected from electronic medical records.

Dyspnea was measured through the mMRC dyspnea scale (with scores between 0–4) before and after a 6 min walking test (6MWT) [28].

Continuous variables were presented as mean ± standard deviation (SD) and categorical variables as frequencies and percentages. Group comparisons for continuous variables were performed using independent sample *t*-tests. Categorical variables were compared using the chi-square test or Fisher’s exact test as appropriate. The interobserver agreement for diaphragm thickness measurements was assessed using Bland–Altman analysis. Receiver operating characteristic (ROC) curve analysis was performed to evaluate the discriminative ability of mean left hemidiaphragm thickness for SSc-ILD group membership; the optimal cut-off was determined using the Youden index, and the area under the ROC curve (AUC) was reported with 95% confidence intervals. Binary logistic regression adjusted for age and sex was performed to assess the independent association of the diaphragm thickness cut-off with group membership; model calibration was evaluated using the Hosmer–Lemeshow test. Cross-tabulation with Mantel–Haenszel testing was used to obtain unadjusted odds ratios. The required sample size for correlation analyses was estimated using the Fisher z transformation method (α = 0.05, two-tailed); with the available cohort of 161 participants; this study had 90% power for r = 0.25. Statistical significance was set at *p* < 0.05. Analyses were performed using IBM SPSS Statistics (version 26) and MedCalc (version 20.106). Of the 69 patients with SSc-ILD, complete functional data (spirometry, DLCO, and 6MWT) were available for 52 patients; the remaining 17 had incomplete records due to unavailability of pulmonary function tests or inability to complete the 6MWT owing to clinical limitations (severe dyspnea or significant exertional desaturation). Diaphragm thickness measurements were available for all 69 patients and were used in all primary comparisons with controls.

## 3. Results

- This study included 161 patients divided in two groups. The first lot was made of 69 patients diagnosed with SSc (36 with the diffuse SSc form and 33 with the limited SSc form). All patients from this lot were previously confirmed with SSc-ILD disease. Out of 69 patients with SSc, 52 had complete data and were further included in the final analyses. Our control lot included 92 age- and gender-matched patients with no previous history of pulmonary diseases. The control group was age- and sex-matched to the initial SSc cohort; subsequent analyses were performed on patients with complete available data. The SSc lot included 60 females and 9 males, with a mean age of 58.9 ± 11.8 years, and the controls included 76 females and 16 males, with a mean age of 57.6 ± 10.3 years. Nutritional status was evaluated through BMI with a mean of 23.01 kg/m^2^. The mean time from diagnosis was 7.5 years. Baseline characteristics are summarized in Table 1.

**Table 1:** Baseline characteristics of patients with systemic sclerosis (SSc).

Variable	Patients with SSc (n = 69)
Age, years	58.9 ± 11.8
Female sex, n (%)	60 (87.0)
Male sex, n (%)	9 (13.0)
Disease duration, years	7.5 ± 6.5
Body mass index, kg/m^2^	23.0 ± 4.2
Diffuse SSc, n (%)	36 (52.2)
Limited SSc, n (%)	33 (47.8)
Anti-Scl70 positive, n (%)	38 (55.1)
Anticentromere positive, n (%)	27 (39.1)
Anti-Ro positive, n (%)	12 (17.4)

Data are presented as mean ± standard deviation or number (percentage).

**Figure 1:**
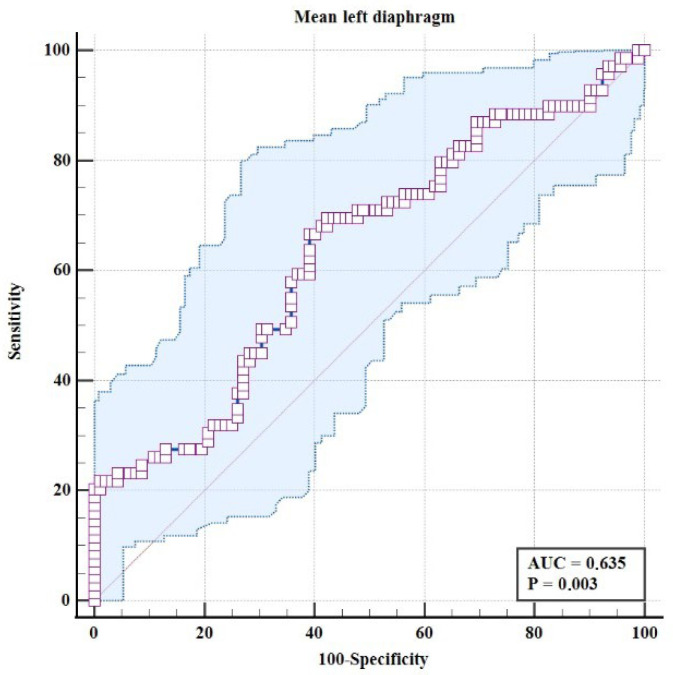
ROC curve for mean left diaphragm thickness (AUC = 0.635, *p* = 0.0025), showing modest but significant discrimination between patients with SSc and controls. Optimal cut-off: ≤3.115 mm (sensitivity 66.7%, specificity 60.9%) *as determined by the Youden index. The red line represents the ROC curve; the blue shaded area indicates the 95% confidence interval. The red box marks the optimal cut-off point.*

**Figure 2:**
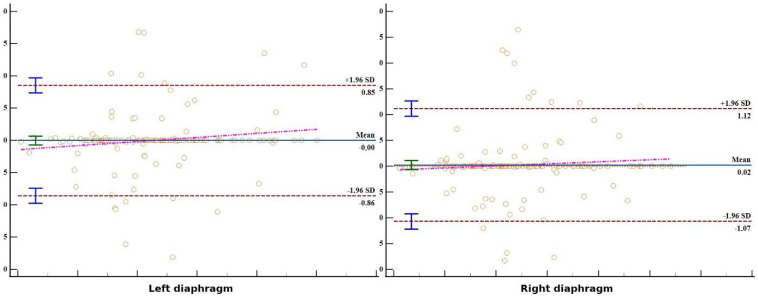
Bland–Altman plot showing the agreement between two measurements of left diaphragm thickness. The *x*-axis represents the mean of thickness measurements 1 and 2, while the *y*-axis shows their difference. *The solid blue line indicates the mean difference (bias); the dashed blue lines represent the 95% limits of agreement (mean ± 1.96 SD). Each circle represents one individual measurement pair.*

- Blood tests showed normal total serum protein levels (7.21 g/dL) and albumin levels as well as normal seric muscle enzyme values, suggesting no overt malnutrition in the studied cohort.
- To evaluate cardio-pulmonary function we performed spirometry, DLCO, 6 min walking test (6MWT) and transthoracic cardiac ultrasound. Looking at the SSc group, almost half of them (37) had a lower FVC (62.92%) and 41 patients presented reduced values of DLCO (60.54%). A total of 18 patients were suspected of PAH at cardiac ultrasound (mean PAPs = 49.5 mmHg). The 6MWT showed reduced performance in 22 patients.
- Receiver operating characteristic (ROC) analysis for the mean thickness of the left diaphragm muscle, assessed by CT at the zone of apposition, revealed an AUC of 0.635 (95% CI: 0.556–0.709, *p* = 0.0025), indicating modest but statistically significant discriminative ability. The optimal cut-off value was ≤3.115 mm, with 66.7% sensitivity and 60.9% specificity; given this limited accuracy, the cut-off should not be used as a standalone diagnostic criterion but may serve as a supplementary indicator within a broader clinical assessment (Figure 1).

- Agreement between the two sets of diaphragm thickness measurements obtained by independent radiologists was assessed using Bland–Altman analysis, which showed a negligible mean difference (−0.002, *p* = 0.95) and limits of agreement of −0.86 to +0.85, indicating no systematic bias. A simple linear regression adjusted for the time interval between the two CT scans showed no significant proportional bias (*p* ≈ 0.06) (Figure 2).

- Group comparison revealed that the left hemidiaphragm (Mean DS) was significantly thinner in patients with SSc-ILD compared with controls (2.98 ± 1.04 mm vs. 3.44 ± 0.97 mm; *p* = 0.004). The right hemidiaphragm (Mean DD) showed a trend toward reduction in patients with SSc that did not reach statistical significance (3.21 ± 1.09 mm vs. 3.52 ± 1.05 mm; *p* = 0.072).
- However, the RD thickness was statistically significant lower in patients with SSc who presented a lower FVC (*p* < 0.05). This indicates a potential concomitant involvement of diaphragmatic muscle in patients with ILD-SSc.
- We also performed a cross-tabulation plus Chi-square analysis and Mantel–Haenszl test. The odds ratio value was like that from binary logistic regression (OR = 3.11). Thus, gender and age do not have an important impact on the predictive value for the chosen muscle thickness cut-off.
- No significant difference was identified in CT-measured diaphragm thickness regarding the autoantibody profile, skin extension or other organ involvement (gastrointestinal tract, heart or kidney). Moreover, the diaphragm thickness does not appear to change according to disease duration (*p* = 0.082). We also tried to find a correlation with the treatment, but the variable of interest is non-dependent on immunosuppressive or antifibrotic agents.

## 4. Discussion

Through this study, we have attempted to determine whether there is a correlation between diaphragmatic morphology changes and the progression or severity of the SSc disease.

Diaphragm thickness was assessed by two different radiologists who analyzed a follow-up CT scan. The results were assessed using Bland–Altman analysis, which showed a negligible mean difference (−0.002, *p* = 0.95) and limits of agreement of −0.86 to +0.85, indicating no systematic bias. A simple linear regression adjusted for the time interval between the two CT scans showed no significant differences between the results obtained in measurements.

Although we were not able to determine multiple correlations between the measurement of the diaphragm thickness and the predictive factors of disease severity, we found a significant decrease in diaphragm thickness in patients with a lower FVC. This assessment may be a useful tool in the future and could be used as a predictive factor for disease progression.

It is known that patients with cardiopulmonary involvement in SSc can present diaphragmatic dysfunction that can lead to the worsening of dyspnea. Further prospective studies are needed to see if measurements change with a proper treatment of the underlying comorbidity and pulmonary rehabilitation [29].

Sarcopenia and a low BMI is a frequent finding in patients with SSc and it is associated with lower muscle strength and mass [30]. Kantarci et al. showed that BMI values < 18.5 and greater than 40 were associated with statistically lower diaphragm mobility in healthy subjects [31]. Even so, a recent meta-analysis that incorporated two studies with a total of 320 patients with evaluation of FVC showed that patients with sarcopenia did not have lower FVC-predicted values than those without sarcopenia, thus there was no significant difference between sarcopenic and non-sarcopenic patients regarding pulmonary function [31].

The mean age in our SSc group was 58.9 ± 11.75 and the controls had a mean age of 57.59 ± 10.26. Analyzing the data, we did not find any predictive correlation between gender and age and cut-off value of muscle thickness in either of the groups. This was also shown in van Doorn et al., who found a constant diaphragm thickness in a broad age range in healthy subjects [32].

The performance of mean left diaphragm thickness in discriminating between patients and controls was modest (AUC 0.635), but the result was statistically significant. This shows that thinning of the diaphragm muscle is associated with lung involvement in systemic sclerosis. A cut-off value of ≤3.115 mm may help distinguish patients with SSc from healthy controls, although with limited accuracy. This likely reflects respiratory muscle weakness due to interstitial lung disease and reduced inspiratory effort. Therefore, diaphragm thickness should not be used alone, but it can provide additional value when combined with pulmonary function tests and HRCT, serving as a simple and non-invasive bedside marker.

Patients with a more severe disease may present with worse SSc-ILD and impaired respiratory muscle function. On the other hand, ILD is associated with increased lung elastic recoil, which in turn can limit diaphragmatic mobility [23]. In addition, an overlap of SSc with other connective tissue diseases—including dermatomyositis, polymyositis, Sjögren’s syndrome, systemic lupus erythematosus, and rheumatoid arthritis—can further influence both the clinical presentation and treatment approach [33]. The present study focused exclusively on patients with SSc; however, future larger prospective studies assessing diaphragmatic function should also include cases with overlapping connective tissue diseases.

**Strengths**

This is among the first studies to investigate diaphragmatic muscle thickness as a potential marker of lung involvement in systemic sclerosis (SSc), offering a new non-invasive perspective.

This study includes both patients with SSc and healthy controls, allowing direct comparison and calculation of cut-off values.

By identifying diaphragmatic thinning in SSc, this study highlights a potential evaluation tool that complements standard pulmonary function tests and imaging.

**Limitations**

The main limitation of this study lies in its retrospective cross-sectional design, which precludes conclusions about causality, disease progression, or prognostic value. A functional assessment of the diaphragm (diaphragmatic excursion, maximum inspiratory pressure, sniff nasal inspiratory pressure) was not performed, which limits our ability to link morphological thinning directly to respiratory dysfunction. In addition, the relatively small sample size (n = 69) may limit the generalizability of the findings. A further limitation is the absence of formal sarcopenia assessments and detailed body composition analyses. Although overt malnutrition was excluded through normal serum protein and albumin levels and cachexia was an explicit exclusion criterion, granular measures such as bioelectrical impedance analysis, grip strength testing, or DXA-derived lean mass were not available. BMI and nutritional data were also not available for the control group, as controls were retrospectively identified from an imaging database where such data are not routinely recorded. Future prospective studies should incorporate these measures to better isolate the contribution of diaphragmatic pathology from generalized muscle deconditioning.

There is no specific point of measurement for the diaphragm thickness. In this study, we used as a reference measurement the celiac axis on both axial and coronal images based on studies published previously [34]. However, recent data suggest that multiple reference points should be used considering the heterogeneous structure of the diaphragm [35,36]. More research may be needed in order to establish reference points in diaphragmatic measurements by combining CT scanning and ultrasonography.

We also need to take into account malnutrition, sarcopenia and body composition, variables that were not thoroughly investigated in our study, through advanced methods like body impedance or indirect calorimetry. Potential further research on patients with SSc could involve measuring the effects of sarcopenia and malnutrition on diaphragm muscle function.

## 5. Conclusions

To our knowledge, this study is one of the very few to use CT-measured thickness of the diaphragm in patients with SSc, providing novel insights.

This multicentric retrospective study is among the first to investigate CT-measured diaphragm thickness in patients with SSc-ILD. Our findings demonstrate that the left hemidiaphragm is significantly thinner in patients with SSc-ILD compared with age- and sex-matched controls (*p* = 0.004), and that right hemidiaphragm thickness correlates with FVC. However, the discriminative performance is modest (AUC 0.635), and the retrospective cross-sectional design precludes conclusions about causality, disease progression, or prognostic value. Diaphragm thickness measurement represents a hypothesis-generating observation that warrants investigation in prospective longitudinal studies incorporating concurrent functional diaphragm assessment.

## Data Availability

The data presented in this study are available on request from the corresponding authors, the data are not publicly available due to institutional ethics approval and patient privacy regulations.

## Abbreviations

The following abbreviations are used in this manuscript:

SSc	Systemic sclerosis
ILD	Interstitial lung disease
SSc-ILD	Systemic sclerosis-associated interstitial lung disease
PAH	Pulmonary arterial hypertension
HRCT	High-resolution computed tomography
LUS	Lung ultrasound
FVC	Forced vital capacity
DLCO	Diffusing capacity for carbon monoxide
BMI	Body mass index
mMRC	Modified Medical Research Council dyspnea scale
mRSS	Modified Rodnan skin score
6MWT	Six-minute walking test
NSIP	Nonspecific interstitial pneumonia
UIP	Usual interstitial pneumonia
OR	Odds ratio
